# Effects of light spectrum on morpho-physiological traits of grafted tomato seedlings

**DOI:** 10.1371/journal.pone.0250210

**Published:** 2021-05-07

**Authors:** Ahmed F. Yousef, Muhammad M. Ali, Hafiz M. Rizwan, Mohamed A. A. Ahmed, Waleed M. Ali, Hazem M. Kalaji, Nabil Elsheery, Jacek Wróbel, Yong Xu, Faxing Chen

**Affiliations:** 1 College of Horticulture, Fujian Agriculture and Forestry University, Fuzhou, China; 2 Department of Horticulture, College of Agriculture, University of Al-Azhar (Branch Assiut), Assiut, Egypt; 3 Plant Production Department (Horticulture—Medicinal and Aromatic Plants), Faculty of Agriculture (Saba Basha), Alexandria University, Alexandria, Egypt; 4 Department of Plant Physiology, Institute of Biology, Warsaw University of Life Sciences SGGW, Warsaw, Poland; 5 Agriculture Botany Department, Faculty of Agriculture, Tanta University, Tanta, Egypt; 6 Department of Bioengineering, West Pomeranian University of Technology in Szczecin, Szczecin, Poland; 7 College of Mechanical and Electronic Engineering, Fujian Agriculture and Forestry University, Fuzhou, China; 8 Institute of Machine Learning and Intelligent Science, Fujian University of Technology, Fuzhou, China; United Arab Emirates University, UNITED ARAB EMIRATES

## Abstract

It is already known that there are many factors responsible for the successful grafting process in plants, including light intensity. However, the influence of the spectrum of light-emitting diodes (LEDs) on this process has almost never been tested. During the pre-grafting process tomato seedlings grew for 30 days under 100 μmol m^-2^ s^-1^ of mixed LEDs (red 70%+ blue 30%). During the post-grafting period, seedlings grew for 20 days under the same light intensity but the lightening source was either red LED, mixed LEDs (red 70% + blue 30%), blue LED or white fluorescent lamps. This was done to determine which light source(s) could better improve seedling quality and increase grafting success. Our results showed that application of red and blue light mixture (R7:B3) caused significant increase in total leaf area, dry weight (total, shoot and root), total chlorophyll/carotenoid ratio, soluble protein and sugar content. Moreover, this light treatment maintained better photosynthetic performance i.e. more effective quantum yield of PSII photochemistry Y(II), better photochemical quenching (qP), and higher electron transport rate (ETR). This can be partially explained by the observed upregulation of gene expression levels of *PsaA* and *PsbA* and the parallel protein expression levels. This in turn could lead to better functioning of the photosynthetic apparatus of tomato seedlings and then to faster production of photoassimilate ready to be translocated to various tissues and organs, including those most in need, i.e., involved in the formation of the graft union.

## 1 Introduction

Vegetable grafting is a popular method used to improve plant health against biotic and abiotic factors and has been implemented in many countries [1, 2]. Successful grafting requires specific environmental conditions during the process (matrix healing and acclimatization), and proper acclimatization is crucial for plants grafted for survival [3].

To ensure the proper healing and acclimatization of the union matrix, it is imperative to control the grafted plants’ micro-climate. In ancient times the traditional method of shading by plastic or organic fiber was used to lower down the temperature and increase the relative humidity around the plants until successful grafting occurs [4, 5]. Although controlling the environmental conditions during grafting of plants is very difficult under normal conditions, many countries e.g., such as China, Japan, and Korea, have developed the acclimatization chambers for the better growth of the union matrix for successful grafting of vegetables. Some researchers have reported fast growth, good survival ratio, and remarkable quality of seedlings grown in healing and acclimatization chambers [6–10].

Non-grafted tomatoes can easily distinguish grafted tomatoes due to their remarkable performance concerning their yield and quality. Grafting can influence their resistance to nematode and soil-borne diseases, uplift fruit yield and quality, and increase its water and nutrient use efficiency [11]. It is reported that the primary purpose of grafting tomatoes is to overcome the yield loss through soil-borne diseases [12]. The significance of grafting includes the plant resistance to biotic and abiotic stresses [13], improvement in crop performance, and maximum use of available resources [14–17].

The light spectrum’s intensity strongly influences plant growth and physiology [18]. Light-emitting diodes (LED) are the fourth generation of a new light source with spectral width (wavelength) of the peak emission of ± 15 and have good spectral characteristics and can be combined to highlight the quality that plants need [19]. It has been proposed as a light emission source for the controlled atmosphere agriculture and spaceflight cultivation systems [20]. LED is a convenient light source as it has a long lifetime, durability, portability, and a different wavelength according to the target. Therefore, it is expected that the LED should be used as an outstanding and effective source of light for controlled atmosphere plantation. Several studies revealed the effects of LED on the growth and development of tomatoes, such as morphogenesis, chlorophyll contents, photochemistry, leaf anatomy, and photosynthesis [21–26].

During drought stress and exposure to light spectrums, the plant produces carbohydrates, proline and auxins, which helps withstand the abiotic stress [27], because of this reason, artificial lights (LEDs) are used to improve the graft-take process of vegetables [28]. For successful grafting of tomatoes, the LEDs can be used as a light source, and the intensity and light period can be controlled easily. In particular, the efficiency of the healing chamber’s space will increase significantly using vertical surface areas.

Over the years, different light conditions have been considered to conduct the physiological studies of photosynthesis. A combination of red and blue LEDs with varying light intensities and wavelengths is considered an effective source for photosynthesis [29]. Tomato seedlings showed higher net photosynthetic rate and maximum quantum yield of photosystem II (Fv/Fm) as compared to control when grown under red: blue (1:1) light [22]. In line with this, Sæbø et al. [30], Shimizu et al. [31], and Kobayashi et al. [32] reported the red light to be significant for photosynthetic apparatus development as it might increase starch accumulation in various plant species by inhibiting the translocation of photosynthates out of the leaves. However, Lactuca sativa plants grown under red LEDs showed lower rates of photosynthesis with a decrease in light intensity [33]. Similar results of the reduced rate of photosynthesis under low light intensity and red LEDs was reported for rice [34] and wheat [35]. Such results may suggest that vulnerability to a lower photosynthetic rate might be linked with changes in multiprotein complexes (PSI and PSII) [33, 36]. Or, it could be attributed to lower leaf nitrogen content because of low chlorophyll and carotenoid content [37].

It is already known that plastid-encoded RNA polymerase regulates the expression of *PsaA* and *PsbA* recruited by a light signal [38]. In addition, artificial light can enhance the expression of *PsaA* and PsbA proteins [24, 39], and *PsbA* protein synthesis and degradation are subject to light regulation [40, 41]. Importantly, deletion of *PsaA* and *PsbA* in tobacco (Nicotiana tabacum cv. Petit Havana) resulted in simultaneous alteration of genes located in both the chloroplast and nucleus [42]. Therefore, the light-dependent regulation of *PsaA* and *PsbA* could help explain the difference in plant growth and development.

In the present study, the effects of light quality on graft-take ratio, some physiological traits and seedling quality of tomato were investigated. In addition, the gene expression performance of two photosynthetic genes (*PsaA* and *PsbA*) was investigated under different optical spectra.

## 2 Material and methods

### 2.1 Plant and environmental conditions

**Fig 1:**
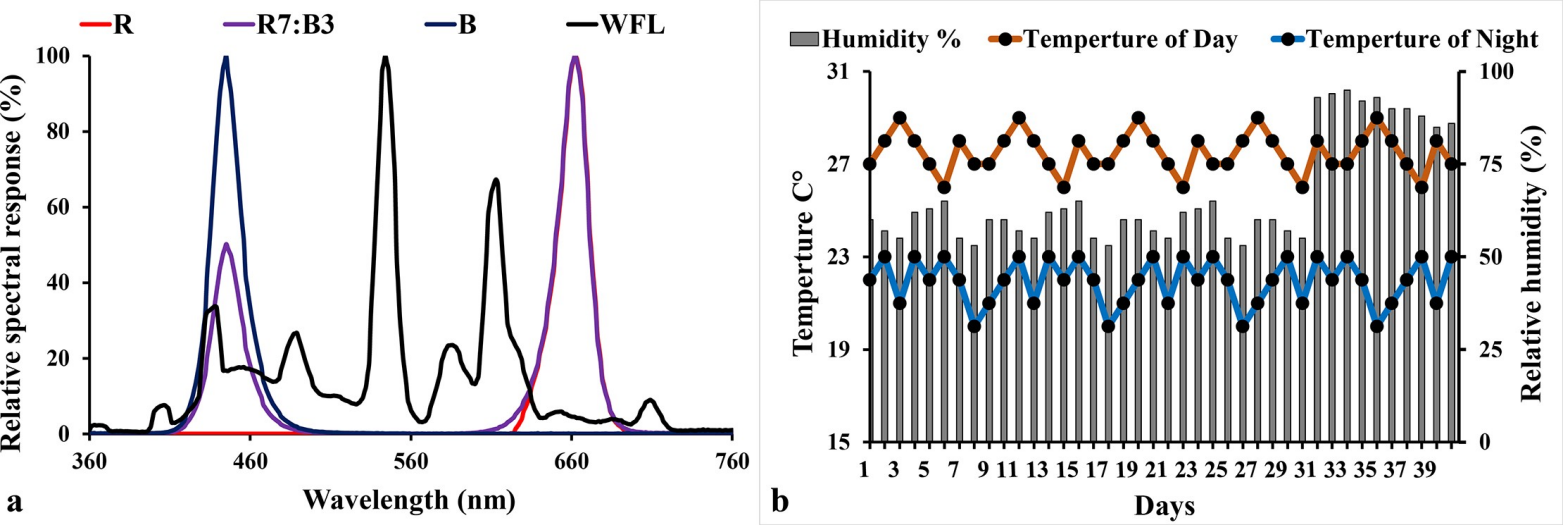
Spectrum distribution of the treatments LED light in the experiment and environmental conditions.

Rootstock cultivar of tomato (*Solanum lycopersicum* L. *var*. *Gangmu No*.*1*(钢木1号)) was resistant to bacterial wilt, resistant to the death of seedlings, Production company: Kaikai 1681 Seeds (Weifang, Shandong Province, China) Co., Ltd. Scion cultivar of tomato (*Solanum lycopersicum* L. *var*. *Millennium* (千禧)) was High yield, Production company: Farmers’ Friends Seedling (China) Co., Ltd. The experimental system includes four (4) treatments; each treatment has 3 chambers (same conditions) as replicates; each chamber has 60 x 60 x 60 cm dimensions. The seeds were planted for 30 days before grafting under (100 μmol m^-2^ s^-1^, ratio of red: blue light was R7:B3) which were chosen as the best LEDs light source in our previous research [43, 44], then the seedlings were grafted and put under the treatments of this study. The seeds of cultivars were sown in 32-cell cells plug trays (28 cm width × 54 cm length × 6 cm height. Luoxi Plastic Products Co., Shandong, China), then after 21 days transplanted in pots (D 7 cm ×H 10cm, Luoxi Plastic Products Co., Shandong, China) that were filled with the commercial growing substrate (N1:P1:K1≥ 3%, Organic matter ≥ 45%, pH 5.5–6.5, Jiangping Enterprise Co., Fujian, China). Environmental conditions in growth chambers are shown in Table 1 and Fig 1. Irrigation was provided for the seedlings as required. Seedlings started to receive fertilization based on water-soluble fertilizers (compound fertilizers "N-P_2_O_5_- K_2_O ≥ 54% 20:20:20+TE", Ruierkang Co., Russia, and Stimufol Amino (compound fertilizers “N 25%, P 16%, K 12%, Amino acids 2%, Bo 0.044%, Fe 0.17%, Mo 0.001%, Zn 0.03%, Cu 0.085, Co0.01%, Mg 0.02%, Mn 0.085% and EDTA” Shoura Co., Egypt.) two times per week through irrigation, one week after sowing.

**Table 1:** Parameters of the LED light properties used in the study.

Treatments		Light spectral ratios	Peak wavelength λp (nm)	Photon flux density (μmol m^-2^ s^-1^)	Duration (hours)	Temperature (°C)		Relative humidity (%)
						Day	Night	
**Pre-grafting**	**R7:B3**	**70:30**	**662**	**100 ±2**	**12**	**27 ± 2**	**23 ± 2**	**60 ± 10**
**Post-grafting**	**R**	**100**	**662**	**100 ±2**	**12**	**27 ± 2**	**23 ± 2**	**90 ± 5**
	**R7:B3**	**70:30**	**662**	**100 ±2**	**12**	**27 ± 2**	**23 ± 2**	**90 ± 5**
	**B**	**100**	**445**	**100 ±2**	**12**	**27 ± 2**	**23 ± 2**	**90 ± 5**
	**WFL**	**100**	**544**	**100 ±2**	**12**	**27 ± 2**	**23 ± 2**	**90 ± 5**

### 2.2 Grafting experiment

The experiment was repeated three times under the same conditions. The rootstock and scion were splice-grafted at 30 days after sowing when rootstock and scion cultivars had 3–4 compound leaves [1]. The grafted seedlings were put in a box (Plastic box for maintaining the humidity) into LED chambers with high humidity of approximately 90–95% with a transparent cover on top. The boxes are suffocating for seedlings, so after three days, the ventilation holes were opened. After three days, the transparent cover was removed for 5 minutes, and the period increases until the seventh day with high humidity of approximately 90%. After seven days, the transparent cover was removed 30 minutes daily with approximately 70–90% humidity. After nine days, the boxes can ventilate for 2–3 h daily with humidity of approximately 70%. After twelve days, the union is completed between rootstock and scion.

### 2.3 Growth and biomass parameter measurements

Growth parameters were estimated 20 days after grafting. Measurement of shoot length was taken from the rhizome base to the plants’ top using a ruler (cm). Stem diameter was measured using digital calipers (mm), and the fresh and dry mass was weighed using an electronic balance (0.0001 g). The total leaf area (cm^2^) (summation of leaf areas) was estimated as described by Pandey and Singh [45]. Fresh shoots and roots were put in paper bags and transferred to a drying oven at 75°C for at least 48 h to obtain the dry weight. Graft-take was estimated using the equation:

Graft‐take%=(Numberofsurvivingseedlings/Numberofgraftedseedlings)×100). (1)

### 2.4 Chlorophyll content measurements

The chlorophyll content was examined 20 days after grafting using 0.2 g of the fresh medium-aged leaves with excluded the edges and veins of leaves. Fresh leaves tissue was cut, ground well, then put in 5 ml 95% ethanol and filtered, and the volume completed up to 25 ml using 95% ethanol. The absorbance readings for Chl *a*, Chl *b*, and Car. were evaluated using a spectrophotometer at three wavelengths, 665, 649, and 470 nm [46], respectively, and the results were calculated using the following formulae:

$$Chl\;a\;\left(mg\;g^{‐1}FW\right)=(13.95OD_{665}-6.88OD_{649})V/200\;W.$$ (2)

$$Chl\;b\;\left(mg\;g^{‐1}\;FW\right)=(24.96OD_{649}-7.32OD_{663})V/200W.$$ (3)

$$Car.\;\left(mg\;g^{‐1}\;FW\right)=(1000OD_{470}-2.05Chl\;a-114.80Chl\;b)V/\left(245\times 200\;W\right).$$ (4)

Where: Chl *a*–chlorophyll *a*, Chl *b*–chlorophyll *b*, Car–carotenoid, V–volume, and W–sample weight.

### 2.5 Biochemical contents

After 20 days of grafting, the fresh medium-aged leaves were selected with excluded the edges and veins of leaves. The fresh leaves were cut in small cutting, and the fresh weight of the samples (0.5 g, 0.5 g, and 0.2 g) was taken to calculate the soluble nitrate content, soluble protein content, and soluble sugar content, respectively. Soluble nitrate contents were determined followingby the method of Cataldo et al. [47]. Soluble protein content was resolved to utilize the Coomassie brilliant blue G250 method, while sugar content was determined using an anthrone colorimetric technique [48]. The absorbance of the extraction solution at 410nm (OD410), 595 nm (OD595), and 630nm (OD630) were estimated using a UV-5100B spectrophotometer (Unico, Shanghai, China), respectively. The biochemical contents were evaluated using the following equations:

$$Soluble\;nitrate\;content\;(mg\;kg^{-1}\;FW)=\left(C\times VT\right)/\left(W\times VS\right).$$ (5)

$$Soluble\;protein\;content\;\left(mg\;g^{‐1}\;FW\right)=\left(C\times VT\right)/\left(VS\times W\times 1000\right).$$ (6)

Solublesugarcontent(%)=(C/VS×VT)/(W×106)×100. (7)

Where C was nitrite (mg g^−1^)/ protein (mg g^-1^) / sugar (%) value from the standard curve, VT total volume of samples extracted (ml), VS taken sample solution (ml), and W fresh leaf weight (g).

### 2.6 Measurement of chlorophyll *a* fluorescence

On the 20^th^ day after grafting (DAG), measurements of chlorophyll fluorescence signals were made using the PAM -2500 chlorophyll fluorometer (Heinz Walz GmbH, Effeltrich, Germany). The fourth leaf from the top of each plant (fully developed leaf) was selected for these measurements. For each experimental variation, measurements were made on 4 leaves from 4 different plants (16 measurements/repeats) grown under standard atmospheric CO_2_ concentrations.

Two protocols were applied to measure the photosynthetic efficiency of the plants. The first (chlorophyll *a* fluorescence induction kinetics) was based on the application of one saturation pulse of red LEDs (8000 μmol m^-2^ s^-1^, 300 ms duration) to determine the minimum (Fo) and the maximum chlorophyll fluorescence (Fm) values, after 30 minutes of dark-acclimation with actinic light similar to the growth irradiance (100 μmol m^-2^ s^-1^). The first recorded signal was performed after 40 seconds of the first measurements. Then 14 successive pulses of the same light intensity were applied at 20-second intervals. The second protocol was based on the performance of the rapid light curve (RLC). The light intensity gradient of the RLC was: 0, 2, 31, 101, 198, 363, 474, 619, 785, 981, and 1160 μmol m^-2^ s^-1^.

The effective quantum yield of PSII photochemistry Y(II), non-photochemical quenching (NPQ), photochemical quenching (qP) Oxborough and Baker [49], and electron transport rate (ETR) [50] values were automatically calculated on the base of the following equations:

Y(II)=(Fm′‐Fs)/Fm′ (8)

qP=(Fm′‐Fs)/(Fm′‐Fo′) (9)

NPQ=(Fm‐Fm′)/(Fm′) (10)

ETR=Y(II)×absorbedPFD×0.5 (11)

Where Fm is maximum chlorophyll fluorescence yield obtained with dark-adapted sample; Fʹo is level of chlorophyll fluorescence yield in a brief interruption of actinic illumination in the presence of far-red illumination; Fʹm is maximum chlorophyll fluorescence yield in illuminated samples; Fs is chlorophyll fluorescence yield during actinic illumination.

### 2.7 Sample collection and RNA isolate RNA

Samples were collected to study gene expressions from leaves 20 days after the grafting process. The samples were immediately frozen in liquid nitrogen and stored at -80 C. For RNA extraction, each frozen sample was ground to a fine powder in a stainless-steel grinder. Total RNA was isolated with TRIzol Reagent, following the manufacturers’ protocol (RNAprep Pure Plant Plus Kit, Tian Biotech (Beijing) Co., Ltd). The RNA quality was assessed using electrophoresis on a 1.5 percent agarose gel. The total RNA concentration was determined by measuring the absorbance ratio (A260/280) ranging from 1.8 to 2.0 was used for quantitative real-time polymerase chain reaction (qRT-PCR) analysis on a Nanodrop (Thermo; Nanodrop 2000, USA).

#### 2.7.1 cDNA synthesis

Total RNA samples of the experimental were reverse transcribed into cDNA using the PrimerScript™ RT reagent kit with gDNA Eraser (Perfect Real Time), following the manufacturers’ instructions (Takara Bio USA, Inc.). The cDNA has been diluted to 2X by RNase free dH_**2**_o.

#### 2.7.2 Quantitative real-time PCR analysis

The transcript data for the *Solanum lycopersicum* genome (release ITAG2.4) retrieved from the JGI-sequenced plant genomes website (**https://phytozome.jgi.doe.gov/pz/portal.html#!info?alias = Org_Slycopersicum**). Expression of 2 DEGs (*PsaA* and *PsbA*) and internal control gene (actin) were measured by relative real-time PCR analysis in a 96-well plate. The annealing temperature was between 59°C and 60°C for qRT-PCR. The amplification was performed in a 15 μL reaction volume containing 7.5 μL of TransStart Tip Green qPCR SuperMix, 0.3 μL of each primer, 5.9μL of RNase free dH_**2**_o, and 1μL of the template cDNA. The qRT-PCR was performed using Lightcycler^**®**^ 96 software 1.1. The primer pairs used for the qRT-PCR quantification analysis were designed using Primer3Plus (**https://primer3plus.com/cgi-bin/dev/primer3plus.cgi**); the primer sequences are listed in Table 2. The PCR preincubation conditions were as follows: 95°C for 30s, The PCR amplification conditions were by 45 cycles of 95°C for 5s, and 60°C for 10s, the PCR melting conditions were as follows: 95°C for 5s, 65°C for 1min, and 95°C for 1s, the cooling conditions were as follows: 50°C for 30s. Fluorescent signals were collected at each polymerization step. Three biological replicates and three technical replicates were used per sample. The different gene expression was calculated by the 2^**-ΔΔCT**^ method [51].

**Table 2:** The primer sequences of (*PsaA* and *PsbA*) and internal control gene (actin).

No.	Code	Gene	Forward primer (5’- 3’)	Reverse primer (5’-3’)	KEGG: Annotation
1	*PsaA*	Solyc06g066640.2.1	GAGGTGGCTCAACTCTTGCT	ACCGAGCTTTGGTGGAAGTT	Photosystem I reaction center W protein, chloroplastic;PSBW;ortholog
2	*PsbA*	Solyc02g011990.1.1	TCACTGCTTGTACCACCACC	CAAGAACAGAAGGGCGGGAT	Photosystem II reaction center W protein, chloroplastic; PSBW;ortholog
3	Reference gene (Actin)		CAAACGAGAATTGCCTTGGT	CTTAACATCCGCACCAACCT	Internal control

### 2.8 Statistical analysis

The study was conducted under a completely randomized design (CRD) with three replicates. Collected data was analyzed for analysis of variance (ANOVA) and Duncans’ multiple range test (DMRT) method for pair-wise comparison of mean values at 5% significance level using analytical software package “SPSS Inc., Chicago, IL, USA”.

## 3 Results

### 3.1 Growth parameters

**Fig 2:**
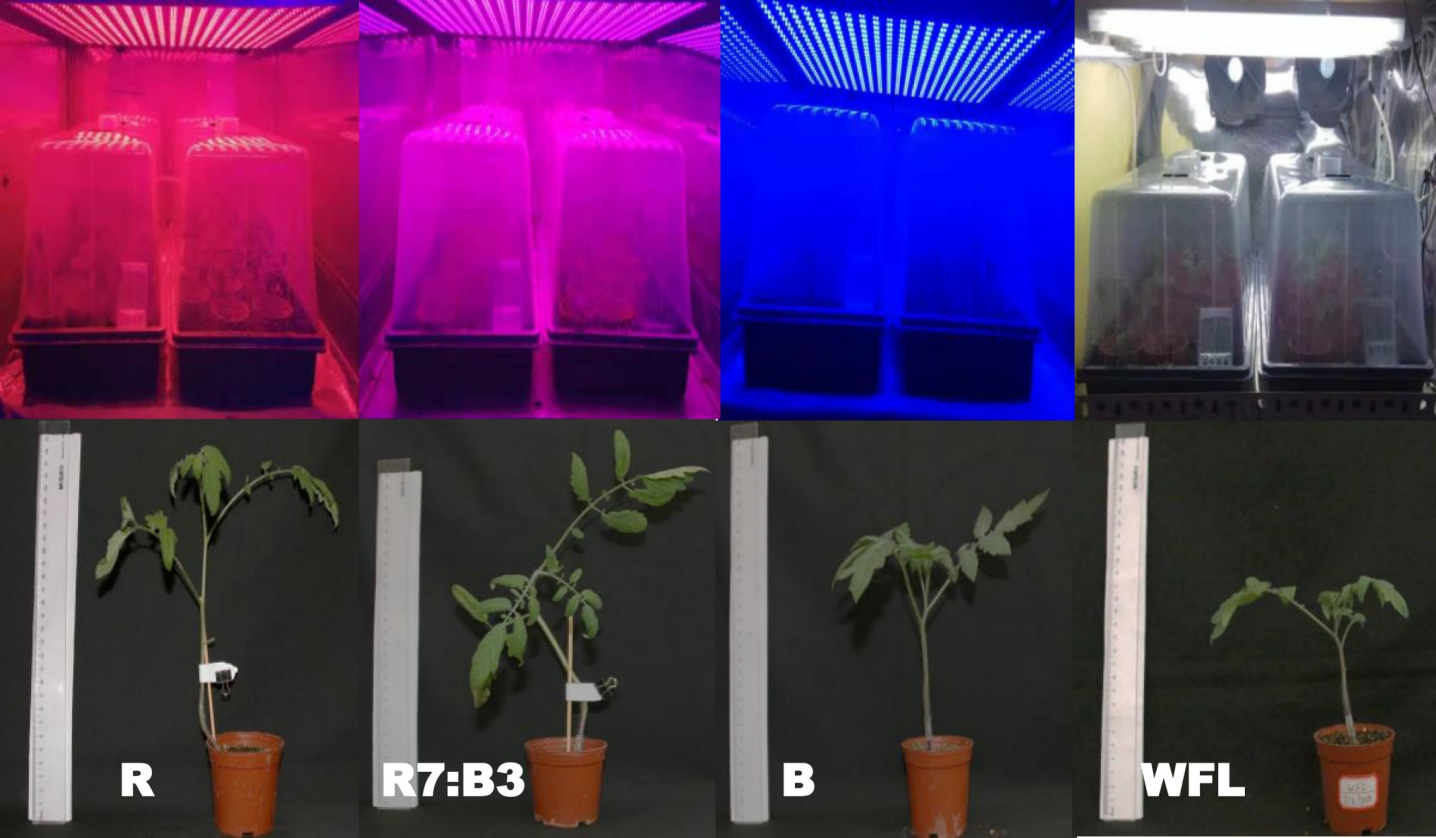
Effect of the LED light on plant morphology of tomato grafted grown under (R = Red light 100%, R7:B3 = Red70%+ Blue30%, B = Blue light 100%, WFL = White Fluorescent Lamps100%) during 20 days after the grafting process.

We can see from (Table 3 and Fig 2) that light ratios with LEDs had significant effects on morphological appearances of grafted tomato seedlings. The shoot length was significantly highest under R treatment, while the lowest shoot length was observed under WFL. The stem diameter of plants below the grafting area was statistically similar, while irradiated under B (4.1 mm) was larger than those under the other LEDs. The plants’ total leaf area with R7:B3 (152.3 cm^2^) was significantly higher than other irradiations with R having the least value. The Root length of plants was statistically similar, while irradiated under WFL (15.2 cm) was longer than those under the other LEDs. Shoot fresh weight under R7:B3 (5.07g) had the highest value, while WFL (3.35g) gave the highest value for Root fresh weight. The highest fresh and dry root weights were observed under R7:B3 (0.62 g and 44.33 mg, respectively). Dry weight content % and Graft-take % under R7:B3 were highest (12.39% and 96.60%, respectively).

**Table 3:** Effect of LED light ratios on growth characteristics of grafted seedlings.

Treatments	Shoot length (cm)	Stem diameter (mm)	Total leaf area (cm^2^)	Root length (cm)	Fresh weight		Dry weight		Dry weight content %	Graft-take %
					Shoot (g.)	Root (g.)	Shoot (g.)	Root (mg.)		
**R**	**28.7±3.53a**	**3.5±0.16a**	**102.7±1.73d**	**13.7±0.88a**	**4.22±0.13c**	**0.22±0.01c**	**0.33±0.01d**	**15.35±0.12d**	**7.78±0.03c**	**60.00±2.02c**
**R7:B3**	**25.2±0.60b**	**3.7±0.29a**	**152.3±1.33a**	**12.6±0.67a**	**5.07±0.04a**	**0.29±0.01b**	**0.62±0.01a**	**44.33±0.19a**	**12.39±0.10a**	**96.60±1.73a**
**B**	**24.3±1.20b**	**4.1±0.27a**	**144.6±2.11b**	**14.7±1.86a**	**4.6±0.09b**	**0.27±0.01b**	**0.53±0.02b**	**31.65±0.38b**	**11.41±0.13b**	**80.40±1.44b**
**WFL**	**21.0±0.58b**	**3.7±0.04a**	**122.1±1.17c**	**15.2±1.59a**	**3.35±0.09d**	**0.35±0.01a**	**0.41±0.01c**	**23.63±0.58c**	**11.72±0.15b**	**81.60±0.87b**

Values are means of four replicates; different letters in the same column indicate significant differences according to DMRT at *P ≤ 0*.*05*. Where R = Red light 100%, R7:B3 = Red70%+ Blue30%, B = Blue light 100%, WFL = White Fluorescent Lamps100%.

### 3.2 Chlorophyll and carotenoid contents, and biochemical contents

The chlorophyll and carotenoid contents of grafted tomato seedlings under different LEDs ratios were shown in Table 4; they appeared to have significant differences. Compared with WFL treatment, the content of Chl *a* was statistically similar under all the treatments, except under R, which had a lower value. The content of Chl *b* under R was higher than those with the other irradiations of LEDs, while R7:B3 treatment showed the lowest Chl b. The carotenoid content in the leaves of R treatment showed higher than those with the other irradiations of LEDs, while B treatment showed the lowest carotenoid. The ratio of chlorophyll *a* to chlorophyll *b* under B was higher than other treatments, while the ratio of chlorophyll *a* to chlorophyll *b* under R treatment was the lowest value. The ratio of total chlorophyll to carotenoids under R7: B3 was higher than the other treatments but did not differ statistically with B, while the ratio of total chlorophyll to carotenoids under R treatment showed the lowest value.

**Table 4:** Effect of LED light ratios on chlorophyll and carotenoid contents, and biochemical contents in grafted seedlings.

Treatment	Chlorophyll *a* (mg g^-1^ FW)	Chlorophyll *b* (mg g^-1^ FW)	Carotenoid (mg g^-1^ FW)	Chlorophyll *a/b* (mg g^-1^ FW)	Total Chlorophyll/ Carotenoid (mg g^-1^ FW)	Nitrate content (mg kg^−1^FW)	Soluble protein content (mg g^−1^FW)	Soluble sugar content (%FW)
**R**	**0.740±0.04b**	**0.666±0.01 a**	**0.445±0.030a**	**0.64±0.06d**	**3.34±0.10c**	**819.050±18.60d**	**6.338±0.07b**	**0.409±0.03c**
**R7:B3**	**0.989±0.03a**	**0.343±0.01 d**	**0.342±0.024bc**	**2.77±0.02b**	**10.30±0.60a**	**1416.700±12.60b**	**10.221±0.17a**	**1.155±0.02a**
**B**	**1.031±0.01a**	**0.420±0.03c**	**0.313±0.010c**	**3.16±0.11a**	**10.28±1.20a**	**1642.900±41.24a**	**9.830±0.12a**	**0.619±0.05b**
**WFL**	**0.980±0.04a**	**0.504±0.03b**	**0.401±0.004ab**	**1.98±0.15c**	**6.51±0.64b**	**1045.200±12.60c**	**10.159±0.23a**	**0.714±0.04b**

Values are means of four replicates ± SE; different letters in the same column indicate significant differences according to DMRT at *P ≤ 0*.*05*. Where R = Red light 100%, R7:B3 = Red70%+ Blue30%, B = Blue light 100%, WFL = White Fluorescent Lamps100%.

Nitrate, soluble protein, and soluble sugar contents under different light ratios in leaves of grafted tomato seedlings are summarized in Table 4. The highest nitrate content was obtained under B. The accumulation of soluble protein content was statistically similar under all the treatments except under R which had a lower value. Furthermore, the concentration of soluble sugar under R7:B3 was the highest among ratios LED light treatments.

### 3.3 Chlorophyll *a* fluorescence characteristic

#### 3.3.1 Measurements under dark-acclimated samples

**Fig 3:**
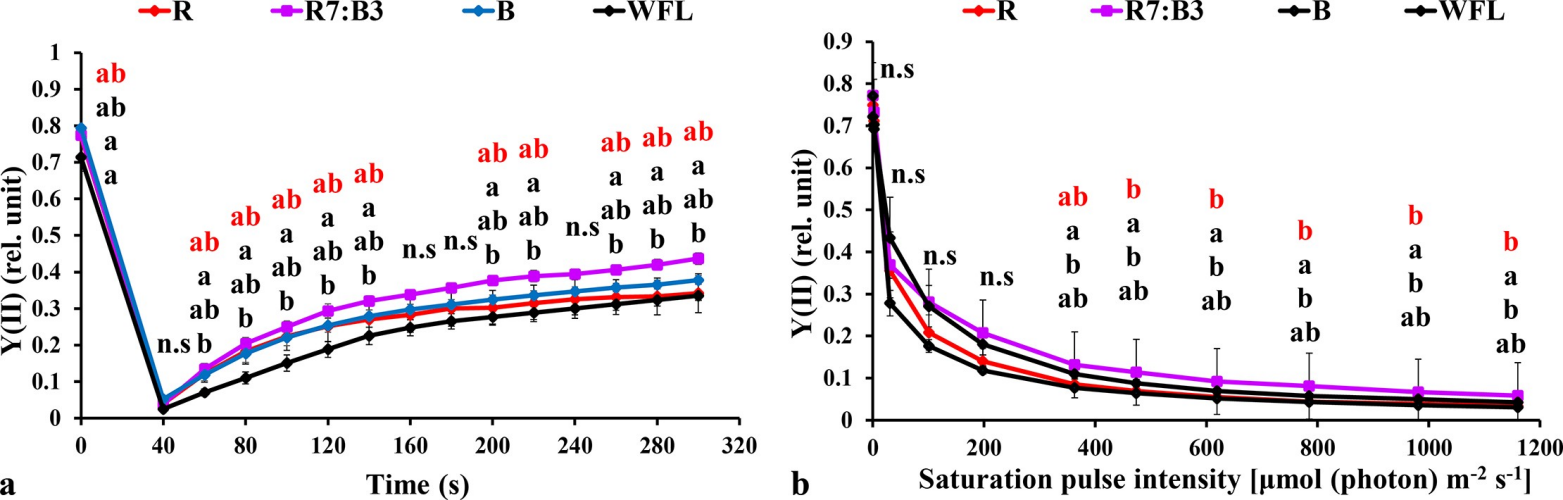
Effect of LEDs light quality on chlorophyll *a* fluorescence induction kinetics of the dark-acclimated **(a)** and RLC of the light-acclimated **(b)** effective quantum yield of PSII photochemistry Y(II) in grafted tomato leaves. Similar letters indicate non-significant difference among treatments according to DMRT at *P* ≤ 0.05. Vertical bars indicate average ± standard error (4 replicates). According to the treatments, sort significance letters from top to bottom (R, R7:B3, B, WFL). Where R = Red light 100%, R7:B3 = Red70%+ Blue30%, B = Blue light 100%, WFL = White Fluorescent Lamps100%.

**Fig 4:**
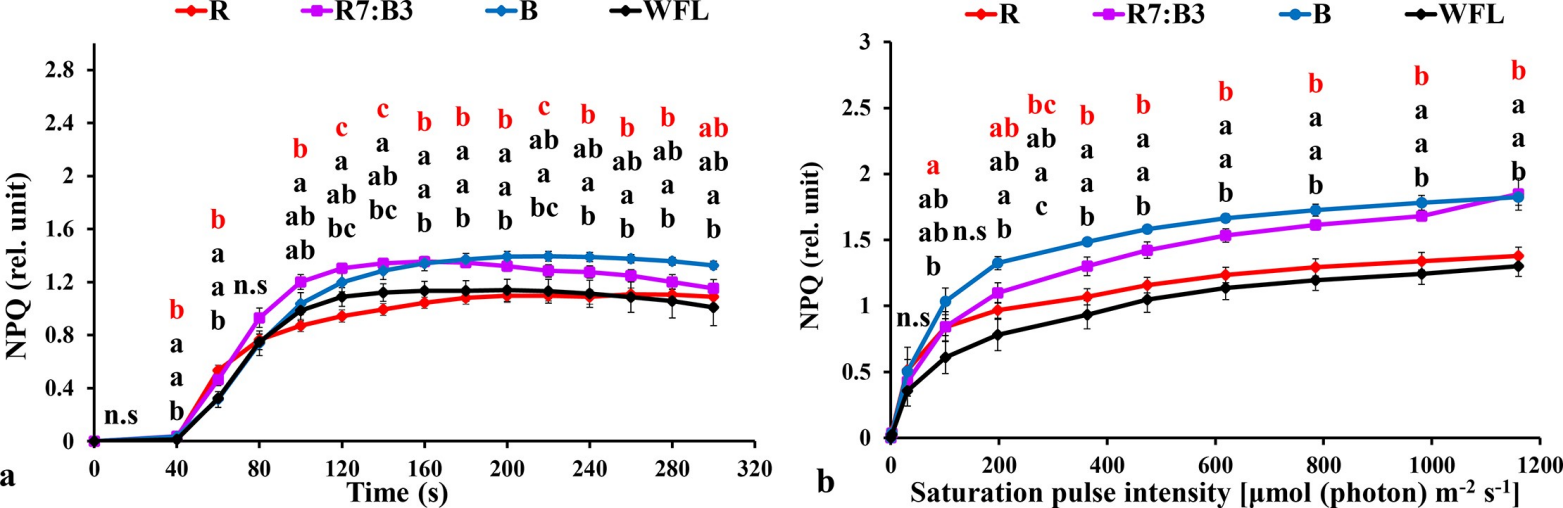
Effect of LED light quality on chlorophyll *a* fluorescence induction kinetics of dark-acclimated (**a**) and RLC of the light-acclimated (**b**) non-photochemical quenching (NPQ) in grafted tomato leaves. Similar letters indicate non-significant difference among treatments according to DMRT at P ≤ 0.05. Vertical bars indicate average ± standard error (4 replicates). According to the treatments, sort significance letters from top to bottom (R, R7:B3, B, WFL). Where R = Red light 100%, R7:B3 = Red70%+ Blue30%, B = Blue light 100%, WFL = White Fluorescent Lamps100%.

**Fig 5:**
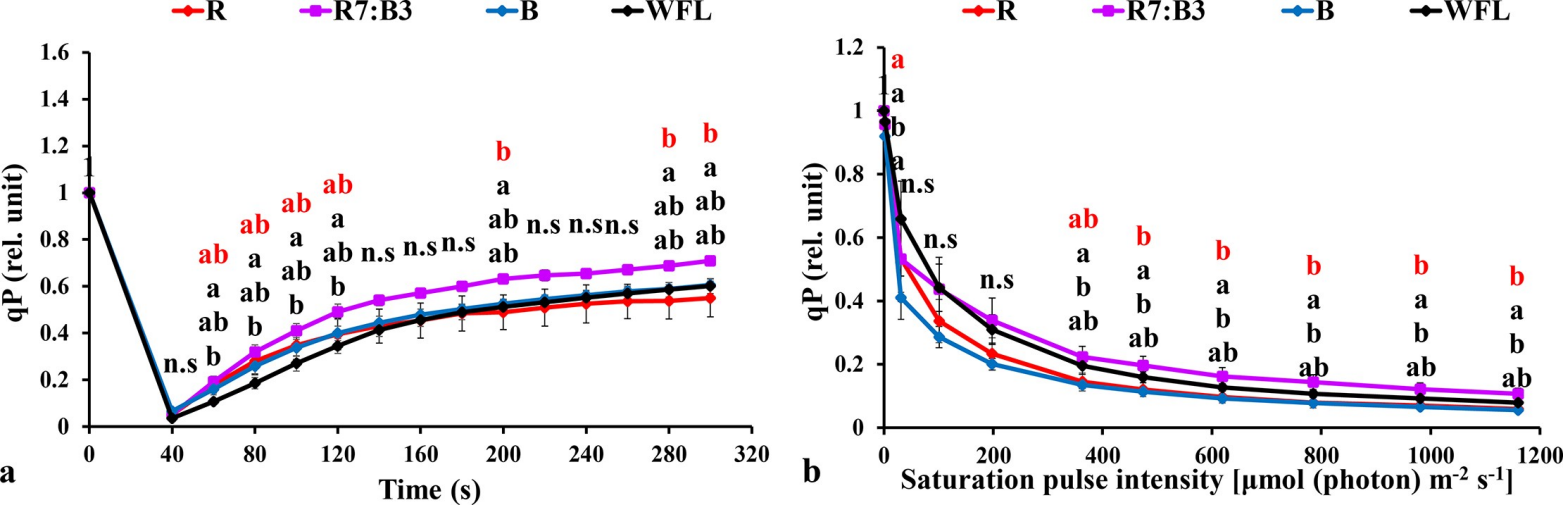
Effect of LED light quality on chlorophyll *a* fluorescence induction kinetics of dark-acclimated (**a**) and RLC of the light-acclimated (**b**) photochemical quenching coefficient (qP) in grafted tomato leaves. Similar letters indicate a non-significant difference among treatments according to DMRT at P ≤ 0.05. Vertical bars indicate average ± standard error (4 replicates). According to the treatments, sort significance letters from top to bottom (R, R7:B3, B, WFL). Where R = Red light 100%, R7:B3 = Red70%+ Blue30%, B = Blue light 100%, WFL = White Fluorescent Lamps100%.

**Fig 6:**
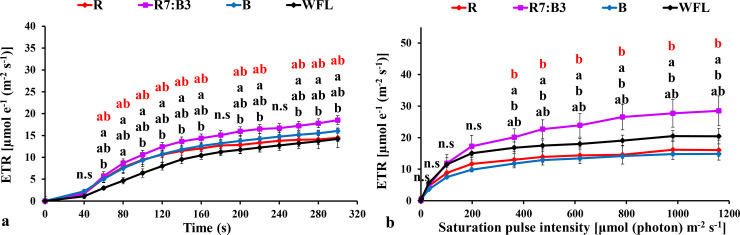
Effects of LED light quality on chlorophyll *a* fluorescence induction kinetics of dark-acclimated (**a**) and RLC of the light-acclimated (**b**) electron transport rate (ETR) in grafted tomato leaves. Similar letters indicate non-significant difference among treatments according to DMRT at *P* ≤ 0.05. Vertical bars indicate average ± standard error (4 replicates). According to the treatments, sort significance letters from top to bottom (R, R7:B3, B, WFL). Where R = Red light 100%, R7:B3 = Red70%+ Blue30%, B = Blue light 100%, WFL = White Fluorescent Lamps100%.

We measured the chlorophyll *a* fluorescence induction kinetics of dark-adapted plants, and the results are shown in Fig 3. The effective quantum yield of PSII photochemistry [Y(II)] decreased directly with the stable light intensity of 100 μmol m^**-2**^ s^**-1**^ at all light spectrums in grafted seedlings, and then increased rapidly with continued exposure to light at all light spectrums. The Y(II) under R7:B3 was significantly higher than other treatments at 60–300 seconds (Fig 3A). Non-photochemical quenching (NPQ) increased rapidly with an increased timeframe in all light qualities in grafted seedlings. The (NPQ) was highest under R7:B3 and B treatments at 40–160 and 180–300 seconds, respectively (Fig 4A). The Photochemical quenching coefficient (qP) decreased directly with a stable light intensity of 1000 μmol m^**-2**^ s^**-1**^ in all light qualities in grafted seedlings then increased rapidly with continued exposure to light at all light qualities. The (qP) was the highest value under R7:B3 at 60–120, 200, and 280–300 seconds (Fig 5A). The electron transfer rate of PSII (ETR) increased rapidly with an increase in exposure time at both light qualities. The ETR performed best under R7:B3 in grafted seedlings at 60–300 seconds (Fig 6A). The ETR there was no significant difference among all treatments except the WFL timeframe at 60, 100–160, 200–220, and 260–300 seconds (Fig 6A).

#### 3.3.2 Measurements under light-acclimated samples

The rapid light curves (RLCs) of light-acclimated photosynthetic quantum yields for PSII were measured. The results showed that the photosynthetic electron transport activity was sensitive to spare light energy and significantly correlated to the oxidation state of electron transfer concatenation. The effective quantum yield of PSII photochemistry [Y(II)] had the best performance under R7:B3 and WFL in grafted seedlings (Fig 3B). It reduced steadily with increasing light intensity at both light quality treatments. The Y(II) under R7:B3 was significantly higher than others at 363–1160 μmol m^**-2**^ s^**-1**^ light intensities except for WFL (Fig 3B).

Non-photochemical quenching (NPQ) had the best performance under B and R7:B3 in grafted seedlings (Fig 4B). The (NPQ) increased gradually with increasing light intensity at all light qualities. The NPQ was significantly higher under B and R7:B3 treatments compared to WFL at almost all light intensities (Fig 4B). The Photochemical quenching coefficient (qP) decreased gradually with increasing light intensity at all light qualities tested. It had the best performance under treatments R7:B3 and WFL in grafted seedlings (Fig 5B). The qP was significantly lower under B and R compared to WFL at almost all light intensities (Fig 5B). The electron transfer rate (ETR) of PSII performed best under R7:B3 in grafted seedlings (Fig 6B), it performed best after light intensity reached 363 μmol m^-2^ s^-1^. The ETR of R7:B3 was significantly higher than other treatments except for WFL, where it was statistically similar, while the ETR of B was significantly lower than other treatments (Fig 6B).

### 3.4 The expression of *PsaA* and *PsbA* genes

**Fig 7:**
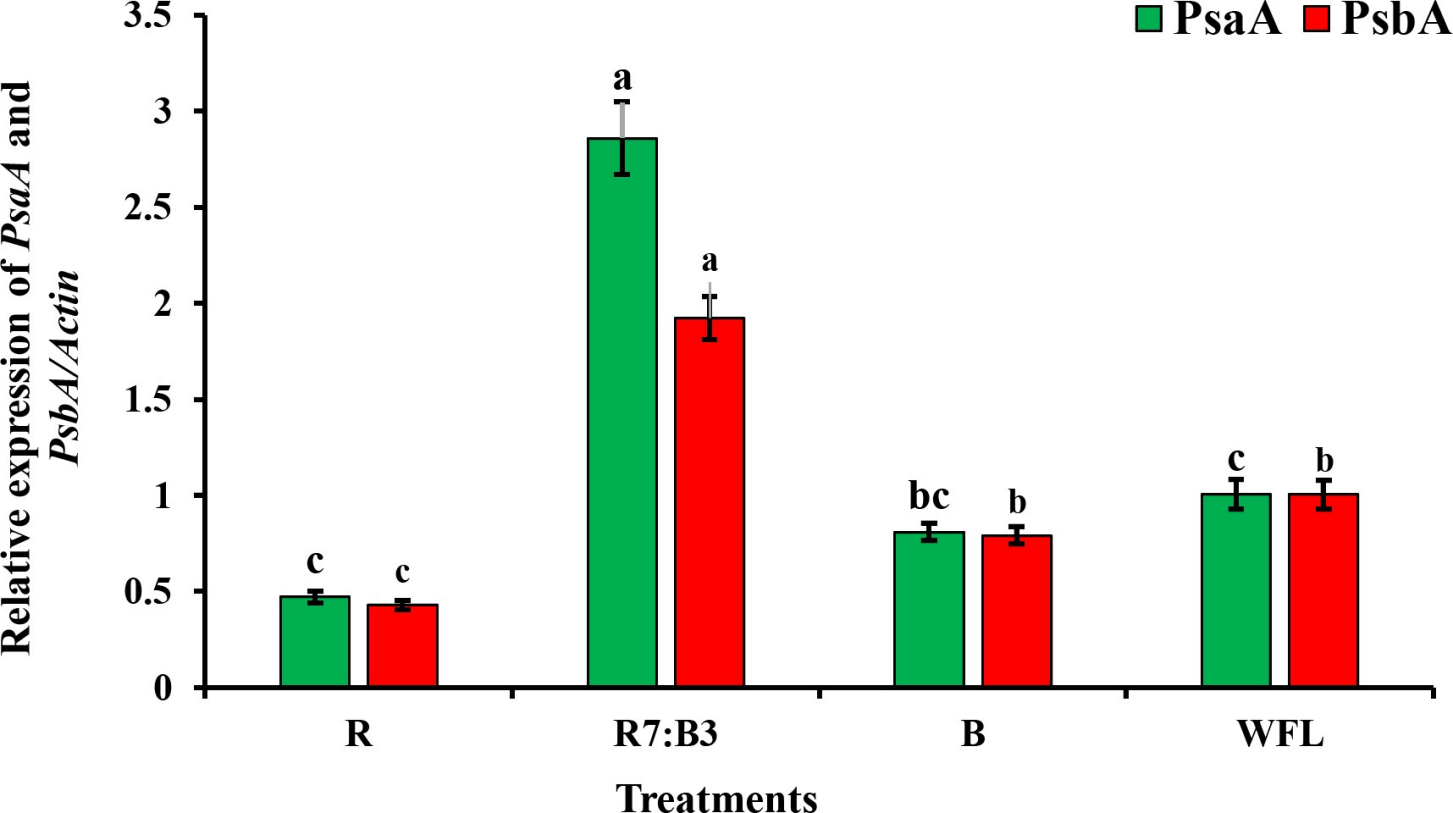
The relative expressions of *PsaA* and *PsbA* in leaves of grafted tomato seedlings. According to DMRT, significant differences among treatments indicate the average ± standard error (P≤0.05) of three technical replicates (n = 4). Where R = Red light 100%, R7:B3 = Red70%+ Blue30%, B = Blue light 100%, WFL = White Fluorescent Lamps100%.

Different sources of artificial light influence the genes encoding the PSⅠ and PSⅠi reaction center proteins (Fig 7). Generally, it was observed that R7:B3 had significant effect on the expression of *PsaA* and *PsbA* when compared to WFL. The relative expressions of *PsaA* and *PsbA* under R7:B3 were 2.86-fold and 1.93-fold, respectively, when compared with WFL. B had no significant effect on the expression of *PsaA* and *PsbA* when compared with WFL. However, a dramatic decrease in expression was observed under R light in *PsaA* and *PsbA*. Particularly, the relative expression of *PsaA* and *PsbA* under R was only 0.47-fold and 0.42-fold, respectively compared with WFL.

### 3.5 Correlations between morphological, biochemical, and physiological parameters

Pearsons’ correlation [52] was carried out among the morphological, biochemical, and physiological parameters observed in this study, as shown in Table 5. There was a significant positive correlation between total leaf area (TLA) and dry weight of shoot and root (R^2^ = 0.984 and 0.956, respectively), shoot fresh weight (SFW) and graft-take (GT) (R^2^ = 0.968), shoot dry weight (SDW) and root dry weight (RDW) (R^2^ = 0.989), root dry weight and qP (R^2^ = 0.965), plant dry weight (PDW) and soluble protein content (Pro.) (R^2^ = 0.991), chlorophyll *a* and soluble protein content (Pro.) (R^2^ = 0.967), soluble sugar content and qP (R^2^ = 0.988), and Y(II) and ETR (R^2^ = 1.000) of grafted seedlings. Whereas, the negative correlations were found among shoot length (SL) and root fresh weight (RFW) (R^2^ = -0.962), chlorophyll *b* (Chl *b*) and total leaf area (TLA) (R^2^ = -0.985), chlorophyll *b* (Chl *b*) and shoot fresh weight (SFW) (R^2^ = -0.971), chlorophyll *b* (Chl *b*) and root fresh weight (RFW) (R^2^ = -0.961), and carotenoid (Car) and nitrate content (Nit) (R^2^ = -0.997).

**Table 5:** Correlation coefficient evaluation between morphological, biochemical, and physiological parameters.

	Morphological parameters										Biochemical parameters						Physiological parameters			
	SL	SD	TLA	RL	FWS	FWR	DWS	DWR	DWC	GT	Chl *a*	Chl *b*	Car	Nit	Pro	Sug	Y(II)	NPQ	qP	ETR
**SL**	1																			
**SD**	-0.41	1																		
**TLA**	-0.35	0.66	1																	
**RL**	-0.608	0.38	-0.288	1																
**FWS**	0.5	0.244	0.635	-0.79	1															
**FWR**	-0.962*	0.185	0.319	0.434	-0.486	1														
**DWS**	-0.237	0.532	0.984*	-0.449	0.72	0.245	1													
**DWR**	-0.239	0.412	0.956*	-0.514	0.697	0.288	.989*	1												
**DWC**	-0.76	0.539	0.855	0.029	0.171	0.764	0.807	0.813	1											
**GT**	0.621	-0.008	0.484	-0.913	0.968*	-0.549	0.605	0.612	0.037	1										
**Chl *a***	-0.765	0.777	0.851	0.229	0.15	0.678	0.758	0.717	0.946	-0.047	1									
**Chl *b***	0.464	-0.592	-.985*	0.256	-0.533	-0.46	-.971*	-0.961*	-0.924	-0.397	-0.883	1								
**Car**	0.342	-0.869	-0.941	0.063	-0.575	-0.219	-0.88	-0.805	-0.756	-0.367	-0.863	0.89	1							
**Nit**	-0.295	0.887	0.919	-0.056	0.59	0.159	0.856	0.773	0.707	0.379	0.833	-0.856	-.997**	1						
**Pro**	-0.827	0.592	0.812	0.16	0.072	0.805	0.742	0.738	0.991**	-0.079	0.967*	-0.882	-0.746	0.699	1					
**Sug**	-0.324	0.088	0.79	-0.548	0.488	0.465	0.853	0.918	0.801	0.481	0.597	-0.855	-0.538	0.484	0.717	1				
**Y(II)**	0.096	0.24	0.846	-0.754	0.874	-0.02	0.925	0.942	0.572	0.839	0.457	-0.818	-0.68	0.66	0.469	0.849	1			
**NPQ**	0.16	0.826	0.602	-0.066	0.66	-0.357	0.546	0.425	0.206	0.467	0.43	-0.463	-0.796	0.838	0.207	0.032	0.447	1		
**qP**	-0.253	0.182	0.856	-0.587	0.603	0.369	0.916	0.965*	0.792	0.575	0.618	-0.896	-0.63	0.585	0.705	0.988*	0.914	0.177	1	
**ETR**	0.1	0.236	0.844	-0.757	0.875	-0.024	0.923	0.941	0.568	0.841	0.453	-0.816	-0.677	0.657	0.465	0.848	1.000**	0.446	0.913	1

* = Correlation is significant at the P ≤ 0.05 level

** = Correlation is significant at the P ≤ 0.01 level, by using Pearson correlation coefficients. SL–Shoot length, SD–Stem diameter, TLA–total leaf area, RL–Root length, FWS–Fresh weight shoot, FWR–Fresh weight root, DWS–Dry weight shoot, DWR–Dry weight root, DWC–Dry weight content, GT–Graft-take, Chl a–Chlorophyll a Chl b–Chlorophyll b Car–Carotenoid, Nit–Nitrate content, Pro–protein content, Sug–sugar content, Y(II)–effective quantum yield of PSII photochemistry Y(II), NPQ–non-photochemical quenching, qP–photochemical quenching coefficient, ETR–electron transport rate.

## 4 Discussion

The quality of the seedlings is essential for the grafted plants to survive, and there are ecological aspects that affect the progress and expansion of the seedling before grafting, such as light spectrum, light intensity, temperature. For photosynthesis, chloroplasts mainly absorb red and blue light [53]. Our study examined the spectra of four frequently utilized light sources and found that grafted tomato seedlings developed well under R7:B3 because R7:B3 had abundant red and blue light. Earlier researches have revealed that red light works on the accumulation of chlorophyll, carotenoid, and anthocyanins [54, 55], and delay flower differentiation and revitalize inter-node elongation [56]. Moreover, red light helps plants resist abiotic and biological stresses [29]. Additionally, red light could assist raise the plant biomass, while blue light could suppress inter-node elongation and lateral shoot growth to prevent excessive growth [57]. In other studies, the root formation of in vitro Anthurium plantlets was progressively induced under red LED lights [58]. Solano et al. [59] reported that exposing pea and watermelon seedlings to red light for 15 minutes gave the highest increase in fresh weight and height, and longer exposure times decreased seedling growth. Here, there were had statistical differences between the exposure of grafted tomato seedlings to different light qualities on the shoot length, where the highest value was under red light.

Most studies indicated that the combination of red and blue light is most effective in promoting plant growth and development. Cucumber Seedlings had higher yields when grown under a mixture of red and blue lights (R5:B5) than when grown under red light [60]. Kim and Hwang confirmed that high quality in plant factory ‘Mini Chal’ tomato (*Solanum lycopersicum* L.) could be acquired under a mixture of blue and red light [23]. Additionally, barrier tissue cells in the leaves were particularly well-developed, and spongy tissue cells were positioned in an organized modality under red+blue [61]. Studies on tomato (*Lycopersicum esculentum*) and *Salvia plebeia* R. Br. showed that a mixture of red and blue light increased the net photosynthetic rate [22, 23], and could also increase the dry weight and leaf area [22, 23, 60, 62]. Wei et al. [24] found that a mixture of blue and red light (light-emitting diode white/red/blue W1R2B2) and Lee et al. [63] found that Light-emitting diode white/red/blue W1R2B1 was the most beneficial for healing and growing grafted tomato seedlings and best suited for the development of vascular bundles and stomatal behaviors. Recent studies have reported the benefits of broad spectra, covering the PAR area such as *AP673L*, on graft-take, and vascular development [57, 63, 64]. Vu et al. [57] reported that the graft-take ratios were under the red and blue light separately (66.7–55.8%, respectively) was low compared to the treatment of natural light (96.7%); this may be due to the natural light containing both the red and blue spectrum. These results strongly support our results as the best graft-take was (96.60%) under R7:B3.

Chlorophyll contents directly influenced photosynthesis process [65, 66], where the chlorophyll content is affected by the light quality [22, 30, 67, 68]. As observed in our study, blue LED light was favorable for chlorophyll *a*, while the red LED light was the best for chlorophyll *b*, and carotenoid (Table 4). These results are supported by Yang et al. [22] in pepper seedlings, Hoffmann et al. [67] in tomato seedlings, and Zheng et al. [68] in three ornamental pot plants. Our results showed that the ratio of total chlorophyll to carotenoids was observed highest under red with blue light among all other treatments in grafted seedlings. The reason might be that the mixture of red and blue light gives enough absorbed light energy causing high Y(II), NPQ, qP, and ETR. Our study proved the importance of a mixture of red and blue light in grafted seedlings in biochemical accumulation. R7:B3 was advantageous for increasing soluble protein and soluble sugar levels.

Similarly, Bian et al. [69] showed that soluble protein levels and soluble sugar were higher in Lettuce under continuing red+ green+ blue (4:1:1) LED light than under other types (red+blue (4:1), red+ green+ blue (1:1:1), and red+blue (4:1) LED light). However, Xiaoying et al. [70] showed that blue LED light was more advantageous in increasing soluble sugar levels in tomato seedlings than (white light, red LED, orange LED, green LED, red and blue LED, and red, blue and green LED). Further, Cui et al. [71] reported that (red+blue) LED light was higher soluble sugar levels in pepper, cucumber, and tomato seedlings than (red, yellow, green, and blue LED light). These results indicated that the light quality affects the accumulation of soluble sugar and protein in vegetable crops and varies among species and cultivars. In the present study, it was observed that under intensity (100±2 μmol m^-2^ s^-1^) and photoperiod (12 h), B LED light was more advantageous in increasing nitrate concentrations than a mixture of red and blue LED light in the grafted tomato seedling. These results have differed from the study of Bian et al. [69], which showed a mixture of red and blue LED light was more advantageous in increasing nitrate concentrations in hydroponically grown Lettuce.

Our results showed that R7:B3 best promotes photosynthesis II. Moreover, seedlings grown in R7:B3 had the best performance for the effective quantum yield of PSII photochemistry Y(II), photochemical quenching coefficient (qP), and electron transport ratio (ETR), while B had the best performance for non-photochemical quenching (NPQ) as they had more ability to adapt to light. This result agrees with the suggestion of Yang et al. [22] in tomato seedlings.

Therefore, the improvement in quality of grafted seedlings under R7: B3 might be due to the up-regulation of *PsaA* and *PsbA*, which is in agreement with the results of Wei et al. [24], who reported that the grafted tomato seedlings were treated with a combination of white + red + blue light (1:2:1); it was the quality amelioration of tomato seedlings that may be due to the up-regulation of *PsbA* and *PsaA*. *PsbA* and *PsaA* are genes of chloroplast encoding the D1 protein of PSII and the P700 apoproteins of PSI, respectively. The expression of *PsaA* and *PsbA* can regulate the plastid-encoded genes [72] and the nuclear genes [42]. Plastid-encoded and nuclear-encoded polymerases are interactional [42, 73–75]. Additionally, Lee et al. [63] reported that light sources from light-emitting diodes (LEDs) combination of R and B light (W1R2B1) might be helpful contributions for stomatal behaviors and developing vascular bundles of the grafted seedlings during the wound healing period. However, the reason for the different extents of up-regulation is yet unknown.

## 5 Conclusion

Our work revealed that many examined physiological treats such as the total leaf area, dry weight (total, shoot, and root), total chlorophyll/carotenoid ratio, soluble protein, and sugar content have changed positively when the red and blue light mixture (R7:B3) was applied. Furthermore, as determined by a higher effective quantum yield of PSII photochemistry Y(II), better photochemical quenching (qP), and higher electron yield, this light treatment increased the photosynthetic performance of the plants. The observed upregulation of the *PsaA* and *PsbA* photosynthetic genes expression levels can help to understand this effect. We assume that the better function of the photosynthetic apparatus of tomato seedlings caused a faster production of photoassimilate, which was ready to be translocated to different tissues and organs, including those that need it most, i.e., those involved in the formation of the graft union. Further research should be conducted to explore the exact genomic mechanism behind the development of the graft junction under the influence of LEDsquality.
